# Hyperbaric hyperoxemia as a risk factor for ventilator-acquired pneumonia?

**DOI:** 10.1371/journal.pone.0253198

**Published:** 2021-06-23

**Authors:** Morgan Caplan, Thibault Duburcq, Anne-Sophie Moreau, Julien Poissy, Saad Nseir, Erika Parmentier-Decrucq

**Affiliations:** Intensive Care Unit and Hyperbaric Center, Lille University Hospital, Lille, France; BronxCare Health System, Affiliated with Icahn School of Medicine at Mount Sinai, UNITED STATES

## Abstract

**Objectives:**

Ventilator-acquired pneumonia (VAP) is the leading cause of serious associated infections in Intensive Care Units (ICU) and is associated with significant morbidity. The use of hyperbaric oxygen therapy (HBOT) in patients on mechanical ventilation may increase exposure to certain risk factors such as hyperoxemia and the need for multiple transfers. The aim of our study was to assess the relationship between HBOT and VAP.

**Method:**

This retrospective observational study was performed from March 2017 to March 2018 in a 10-bed ICU using HBOT. All patients receiving mechanical ventilation (MV) for more than 48 hours were eligible. VAP was defined using clinical and radiological criteria. Data collection was carried out via digital medical records. Risk factors for VAP were determined by univariate and multivariate analysis.

**Results:**

Forty-two (23%) of the 182 patients enrolled developed at least one episode of VAP. One hundred and twenty-four (68%) patients received HBOT. The incidence rate of VAP was 34 per 1000 ventilator days. The occurrence of VAP was significantly associated with immunosuppression (*p<0*.*029*), MV duration (5 [3–7] vs 8 [5–11.5] days, *p<0*.*0001*), length of stay (8 [5–13] vs 19.5 [13–32] days, *p<0*.*0001*), reintubation (*p<0*.*0001*), intra-hospital transport (*p = 0*.*001*), use of paralytic agents (*p = 0*.*013*), tracheotomy (*p = 0*.*003*) and prone position (*p = 0*.*003*). The use of HBOT was not associated with the occurrence of VAP. Multivariate analysis identified reintubation (OR: 8.3 [2.6–26.6]; *p<0*.*0001*), intra-hospital transport (OR: 3.5 [1.3–9.2]; *p = 0*.*011*) and the use of paralytic agents (OR: 3.3 [1.3–8.4]; *p = 0*.*014*) as independent risk factors for VAP.

**Conclusion:**

Known risk factors for VAP are to be found within our ICU population. HBOT, however, is not an extra risk factor for VAP within this group. Further experimental and clinical investigations are needed to understand the impact of HBOT on the occurrence of VAP and on physiological microbiome.

## Background

Oxygen therapy is a basic and usual form of treatment in critical care. It is supposed to prevent hypoxia and improve the distribution of oxygen in tissues. However, oxygen therapy can cause harmful effects and evidence from recent studies suggests an association between exposure to hyperoxemia and worsening prognosis in different situations [1–3]. Even moderate hyperoxemia (arterial partial pressure of oxygen (PaO_2_) > 100 mmHg) has been associated with increased mortality [4]. In a recent meta-analysis, involving more than 16 000 intensive care unit (ICU) patients, a liberal oxygen therapy strategy (measured by any one of the following: fraction of inspired oxygen (FiO₂), PaO₂, arterial oxygen saturation or peripheral oxygen saturation) was compared to a conservative strategy (with a low oxygen therapy target). Patients treated liberally with oxygen had a dose-dependent increased risk of short-term and long-term mortality [5].

In critically ill patients, exposure to hyperoxemia leads to a variety of consequences such as alteration of muco-ciliary clearance and cell immunity, decrease in surfactant synthesis, hyaline membrane formation, denitrogenation which promotes atelectasis [6, 7]. These can cause hyperoxic acute lung injury and contribute to the occurrence of ventilator-acquired pneumonia (VAP) [8]. But this relationship remains controversial and counter-balanced in certain populations for the sake of security [9]. Finally, there is no general agreement on defining hypoxemia and measurement methods, thus results prove difficult to compare and interpret [10]. This is also true for hyperoxemia.

These studies lead us to question the impact of hyperbaric oxygen therapy (HBOT) on the incidence of VAP in our unit where we perform 2,500 HBOT sessions each year on intensive care patients, half of whom are ventilated and intubated. HBOT can be used safely in the ICU and improve the prognosis of specific pathologies [11]. It involves providing oxygen by means of the airways at a higher than atmospheric level of pressure. This increases oxygen pressure levels in the tissues which causes a number of physiological effects such as an increase in arterial oxygen content, vascular effects of the hyperoxic vasoconstriction induced, the most important effect of which is the reducing of oedema, also infection fighting and healing promoting effects [12–15]. There can be some adverse effects such as barotrauma (usual in ENT, rarely pulmonary) and hyperoxia (Paul Bert effect) causing convulsions which cease as soon as treatment is stopped [15, 16]. Transportation to the hyperbaric chamber is a major difficulty for ICU patients. Nowadays, hyperbaric units working with ICUs are able to keep providing most of the care required for these patients within the hyperbaric chambers. HBOT can cause a huge increase in certain risk factors of VAP such as hyperoxemia [8], transportation, possible increase in the duration of sedation or mechanical ventilation (MV) [15]. Moreover, our patients spend several hours a day in a hyperbaric chamber subjected to supraphysiological oxygen pressures with transient hyperoxemia.

Because reducing risk exposure is a priority, the aim of our study was to determine whether HBOT sessions are a risk factor for the occurrence of VAP in our ICU patients.

## Method

We conducted a retrospective single-center study in a 10-bed intensive care unit located in the University Hospital of Lille, France. All adult patients requiring invasive MV for more than 48 hours from March 2017 to March 2018 were enrolled. This observational study was conducted in compliance with the French law of August 9th 2004 and with the additional law #2012–300 of March 5th 2012. This kind of observational study did not require any prior authorization as mentioned by the above-mentioned laws. The study received approval of the French Data Protection Authority (Commission nationale de l’informatique et des libertés: CNIL) under the reference DEC20-039 and was examined and approved by our Ethical Committee (Comité de Protection des Personnes Nord Ouest IV) under the reference HP 21/01. Patients (or/and families) were informed that their medical records would be used in medical research. Patients (or / and their families) were able to withdraw by simple oral or written request.

### Data collection

The following data were recorded upon admission: age, gender, severity of the illness with sepsis-related organ failure assessment (SOFA) and simplified acute physiology score (SAPS) II, whether admission was surgical or medical, comorbidities (chronic obstruction pulmonary disease (COPD), diabetes, chronic heart or renal failure, cirrhosis, hematological disease, immunosuppression or neoplasia), prior use of antibiotics and reason for admission to the ICU. Known risk factors of VAP were collected from digital medical records (duration of sedation and duration of mechanical ventilation, use and duration of neuromuscular-blockers, stress ulcer prophylaxis, reintubation, prone position, tracheotomy, red blood cell transfusion, intra-hospital transport, number of days with PaO_2_ > 120 mmHg). Many different tracheal tubes were used on our patients, as many patients arrived to the ICU already intubated. When the patient was reintubated during his stay, mostly a tube with subglottic aspiration was used; often a different one from the tube used initially. This is the reason why this data did not seem relevant for analysis here. Finally, survival data was collected either using digital medical records or by calling the referring physician if the patient had been discharged from the hospital (ICU mortality and 28-day mortality).

### Definitions

VAP was defined as the presence or progression of a new radiologic pulmonary infiltrate combined with at least two of the following criteria: fever (≥38.5°C) or hypothermia (≤36°C), hyperleukocytosis (≥10 000/mm3) or leukopenia (≤3500/mm3) and purulent respiratory secretions. For every patient, microbiological samples were taken either by tracheal aspiration (threshold ≥10^5^ CFU / mL) or bronchoalveolar lavage (≥10^3^ CFU / ml). Only the first episode of VAP was taken into account. VAP was described as early if it occurred before the fifth day after intubation. Immunosuppression was defined by the presence of active solid neoplasia or hematological malignancy, corticosteroid therapy (≥1 mg/kg/day or long term >1 month), uncontrolled HIV infection or neutropenia (<500 neutrophils/μL). Prior antibiotic was considered for the 3 months before ICU admission. We consider a day with hyperoxemia as a day with PaO_2_ >120 mmHg on blood gases without control by another blood gas showing normoxemia within the twelve next hours. Intrahospital transport referred to a patient being moved for patient management (radiological or therapeutic procedures) with MV other than being moved into the hyperbaric chamber. Septic shock was defined using the recent criteria of the third international consensus conference on sepsis (SEPSIS-3) [17].

### Study population

Our patients included standard critical patients and patients who were provided with HBOT. HBOT indications followed the recommendations of the 10^th^ European Consensus Conference of Hyperbaric Medicine [18]. HBOT sessions were performed in a multi-place hyperbaric chamber. Obviously, each patient was accompanied by at least one ICU nurse qualified to work in hyperbaric conditions. The HBOT protocol used in our center involved a 15-minute pressure rise from 1 to 2.5 ATA, maintaining this pressure during 90 minutes, followed by a 15-minute decompression period to return to 1 ATA. Only for air embolism did the first session differ, with a maximum pressure of 4 ATA provided for 5 minutes followed by a long plateau at a pressure of 2.8 ATA.

VAP prevention is ensured by limiting exposure to known risk factors. Sedation is suspended as soon as possible, to allow a daily free ventilation test. Patients are in a semi-recumbent position, giving them the benefit of an early chair setting, respiratory and muscular physiotherapy. Our ICU beds continuously measure the elevation of the head of the bed. This information is valuable because it is well-documented that clinicians overestimate the degree of elevation [19]. A minimum positive end-of-expiration pressure of 5cmH_2_O is used. Endotracheal aspirations are performed whenever necessary. All the respirator circuits and filters are not changed on a daily basis, only when soiled, as recommended [20]. The tracheal tube cuff pressure is monitored three times a day. Whenever HBOT is provided, they are filled with water during the actual treatment. Chlorhexidine oral and dental care are performed on a regular basis. Enteral nutrition is preferred, which is why we can usually avoid giving treatment against stress ulcers. No oral or intravenous decontamination is performed. Moving our patients requires specially equipped trolleys or beds to ensure that patient monitoring and treatment are maintained during transfer within a transfer management plan following the guidelines of the Society of Critical Care Medicine [21].

Antibiotic treatment for patients with suspected VAP was based on American Thoracic Society/Infectious Diseases Society of America (ATS/IDSA) and French Intensive Care Society (SRLF) guidelines. For other infections including those of the soft tissues, antibiotherapy was based on written local protocols adapted from international guidelines.

### Statistical analysis

In order to assess risk factors associated with VAP, patients who developed VAP were compared to those who did not, using bivariate and multivariate analyses. The distribution of quantitative variables was tested by the Kolmogornov-Smirnov normality test. Very little data had a statistically normal distribution. To keep things simple, we therefore decided to describe all results of continuous variables as median and interquartile (IQR). Student’s t test or the Mann-Whitney U test was used to compare normally distributed, and skewed continuous variables, respectively. For categorical variables, results are expressed as numbers and frequency (%). Categorical variables were compared using the chi-square (χ2) test or Fischer’s exact test. Differences were considered significant if *p* values were <0.05. Data from univariate analysis with *p* values <0.1 were included in the multivariate logistic regression model using stepwise backward elimination. Statistical analyses were performed using SPSS software (version 22.0; SPSS, Chicago, IL).

## Results

Over the period of our study, 325 patients were admitted to the ICU, 182 required invasive MV for 48 hours or more, 120 of whom were male (66%). Demographic and clinical details of the entire population on admission are shown in **Table 1**.

**Table 1:** Patients included in study: Data upon ICU admission.

	Patients (n = 182)
Age (*years*)	60 [47–68]
Male gender	120 (66%)
BMI (*kg*.*m*^*-2*^)	28 [24–35]
**Severity scores**	
SOFA	7 [5–10]
SAPS II	59 [45–71]
**Admission category**	
Surgical	65 (36%)
Medical	117 (64%)
Prior antibiotic therapy	33 (18%)
Hyperbaric oxygen therapy	124 (68%)
**Chronic diseases**	
Diabetes	61 (34%)
COPD	32 (18%)
Heart failure	26 (14%)
Kidney failure	17 (9%)
Cirrhosis	10 (5%)
Hematological disease	9 (5%)
Immunosuppression	17 (9%)
Neoplasia	23 (13%)
**Cause for ICU admission**	
Shock	86 (47%)
Septic shock	73 (40%)
Cellulitis	63 (35%)
Respiratory failure	28 (15%)
Neurological failure	36 (20%)
Congestive heart failure	5 (3%)
Cardiac arrest	38 (21%)
Other	22 (12%)

Results are expressed in numbers (%) for the categorical variables and in median (IQR) or mean ± standard deviation for quantitative variables. ICU: intensive care unit, BMI: body mass index, SOFA: sequential organ failure assessment, SAPS II: simplified acute physiology score, COPD: chronic obstructive pulmonary disease.

Of the 182 patients, 42 (23%) developed at least one episode of VAP including 9 early VAP (21%). VAP occurred in 16 (28%) patients in the standard care group vs 26 (21%) in the HBOT group. The incidence rate of VAP was 34 per 1000 MV days. The median (IQR) length of time between starting MV until VAP diagnosis was 8 [5–11.5] days. Specific data gathered on admission about patients with VAP as compared with those without VAP can be found in the additional table. On admission, only patients with COPD or with immunosuppression showed significant differences where VAP occurrence was concerned. The results of the univariate analysis of risk factors for VAP are shown in **Table 2**.

**Table 2:** Data on patients with / without VAP.

	No VAP (n = 140)	VAP (n = 42)	*p*
Male gender	88 (63%)	32 (76%)	0.11
Age (*years*)	59.5 [46–69]	60 [49–67]	0.94
**Severity scores**			
SOFA	7 [5–9]	8 [5–11]	0.07
SAPS II	59 [45–70]	61 [44–72]	0.56
Prior antibiotic therapy	26 (19%)	7 (17%)	0.78
**Data regarding ICU stay**			
Stress ulcer prophylaxis	75 (54%)	21 (50%)	0.68
Intrahospital transport	69 (49%)	33 (79%)	**0.001**
Red blood cell transfusion	40 (29%)	21 (50%)	**0.01**
Neuromuscular blockers	30 (21%)	17 (41%)	**0.013**
Length of stay (*days*)	8 [5–13]	20 [13–32]	**<0.0001**
Length of sedation (*days*)	3 [2–4]	5 [3–8]	**<0.0001**
ICU mortality	53 (38%)	20 (47%)	0.26
28-Day mortality	54 (39%)	16 (38%)	0.96
**Ventilation data**			
MV time to VAP (*days*)	5 [3–7]	8 [5–11,5]	**<0.0001**
Tracheotomy	1 (1%)	5 (12%)	**0.003**
Reintubation	10 (7%)	16 (38%)	**<0.0001**
Prone positioning	1 (1%)	5 (12%)	**0.003**
**HBOT**	98 (70%)	26 (62%)	0.32

Results are expressed in numbers (%) for categorical variables and in median (IQR) for quantitative variables. Exposure to risk factors was collected until the onset of VAP or until extubation. VAP: ventilation acquired pneumonia, SOFA: sequential organ failure assessment, SAPS II: simplified acute physiological score, ICU: intensive care unit, MV: mechanical ventilation, HBOT, hyperbaric oxygen therapy.

In our model of logistic regression, we included the different statistically significant variables in univariate: SOFA score, duration of MV, COPD, immunosuppression, neuromuscular blockers, prone position or tracheotomy during hospitalization, reintubation and intra-hospital transport. Reintubation (OR: 8.3 [2.6–26.6]; *p<0*.*0001*), intra-hospital transport (OR: 3.5 [1.3–9.2]; *p = 0*.*011*), use of paralytic agents (OR: 3.3 [1.3–8.4]; *p = 0*.*014*) are the factors associated with the occurrence of VAP. A history of lung disease appeared as a protective factor (0.10 [0.02–0.52], *p = 0*.*006*). HBOT was added to the model in a second multivariate analysis to check that there was no effect on the occurrence of VAP. This was confirmed by the second analysis. The results of the multivariate analysis of risk factors for VAP are shown in **Table 3.**

**Table 3:** Multivariate analysis of risk factors of VAP.

			*Univariate*	*Multivariate*	
	No VAP (n = 140)	VAP (n = 42)	*p*	*OR*	*p*
**Severity scores**					
SOFA	7 [5–9]	8 [5–11]	0.07		0.2
**Chronic diseases**					
COPD	29 (21%)	3 (7%)	0.043	0.1 [0.02–0.52]	**0.006**
Immunosuppression	9 (6%)	8 (19%)	0.029		0.09
**Data regarding ICU stay**					
Intrahospital transport	69 (49%)	33 (79%)	**0.001**	3.5 [1.3–9.2]	**0.011**
Red blood cell transfusion	40 (29%)	21 (50%)	**0.01**		0.3
Use of paralytic agents	30 (21%)	17 (41%)	**0.013**	3.3 [1.3–8.4]	**0.014**
**Ventilation data**					
Duration of MV (*days*)	5 [3–7]	8 [5–11,5]	**<0.0001**		0.2
Tracheotomy	1 (1%)	5 (12%)	**0.003**		0.075
Reintubation	10 (7%)	16 (38%)	**<0.0001**	8.3 [2.6–26.6]	**<0.0001**
Prone positioning	1 (1%)	5 (12%)	**0.003**		0.2
**HBOT**	98 (70%)	26 (62%)	0.32		0.8

Results are expressed in numbers (%) for categorical variables and in median (IQR) for quantitative variables. Results of multivariate analysis were reported as odds ratios (OR), and statistical significance was ascertained by the 95% confidence interval. VAP: ventilation acquired pneumonia, SOFA: sequential organ failure assessment, COPD: chronic obstructive pulmonary disease, ICU: intensive care unit, MV: mechanical ventilation, HBOT: hyperbaric oxygen therapy.

Within our cohort, 124 (68%) patients were provided with HBOT. There were significant differences between both groups. Patients without HBOT appeared more immunocompromised (10 (17%) vs 7 (6%); *p = 0*.*012*) and suffered more from COPD (20 (35%) vs 12 (10%), *p<0*.*0001*) than the population provided with HBOT. A higher rate of surgical admissions was noted in the HBOT group, 2 (3%) versus 63 (51%), *p<0*.*0001*). On admission, the group without HBOT was more severe on both severity scores, SAPS II (62.5 [48–76] vs 59 [43–68], *p = 0*.*032*) and SOFA (7.5 [5–11] vs 7 [5–9], *p = 0*.*044*), and older (63.5 [54–71] vs 57 [43–65] years, *p = 0*.*001*). The specific data about the ICU stay for populations with and without HBOT is shown in **Table 4.** VAP occurred in 16 patients (28%) in the standard care group vs 26 (21%) in the HBOT group, with no statistically significant difference.

**Table 4:** Patient data during ICU stay without / with HBOT.

	without HBOT (n = 58)	HBOT (n = 124)	*p*
Stress ulcer prophylaxis	37 (64%)	59 (48%)	**0.041**
Red blood cell transfusion	17 (29%)	44 (36%)	0.41
Prone positioning	4 (7%)	2 (2%)	0.083
Tracheotomy	2 (3%)	4 (3%)	1
Neuromuscular blockers use	16 (28%)	31 (25%)	0.71
Length of ICU stay (*days*)	9.5 [6–18]	10 [6–18]	0.93
Total duration of MV (*days*)	5 [4–8]	5 [4–8]	0.75
Duration of sedation (*days*)	3 [2–5]	3 [2–5]	0.29
ICU mortality	28 (48%)	45 (36%)	0.12
28-day mortality	28 (48%)	42 (34%)	0.063

Results are expressed in numbers (%) for categorical variables and in median (IQR) or mean ± standard deviation for quantitative variables. Exposure to risk factors was collected until the onset of VAP or until extubation. ICU: Intensive Care Unit, HBOT: hyperbaric oxygen therapy, MV: mechanical ventilation.

HBOT was indicated for an infectious reason (cellulitis) in 68 patients (55%) with a median of 11 sessions, for cerebral anoxia after self-attempted hanging in 38 patients (31%) who were all provided with 5 sessions, for air embolism in 11 patients (9%) with a median of 4 sessions, for carbon monoxide poisoning with a median of 2 sessions and for one patient whose indication was severe limb trauma who underwent a total of 22 sessions. VAP were polymicrobial in 9 (21%) patients and related to multiple drug resistant (MDR) bacteria in 10 (24%) patients. In 4 cases, medical treatment was withheld, so no analysis was performed at to the pathogen involved. Gram-negative bacteria represent 78% of all cases. *K*.*pneumoniae* (17%), *S*.*aureus* (15%) and *P*.*aeruginosa* (13%) were the most frequently identified bacteria (**S1 and S2 Tables**).

An analysis performed on the subgroup of patients without HBOT found a significant association between the occurrence of VAP and the number of days with hyperoxemia (1 day [0–2] in the group without VAP vs 2 [1–3] in the group with VAP, *p* = 0.034). A further analysis on this same sub-group comparing the percentage of days with hyperoxemia to the days with MV (20% [0%-38%] vs 25% [10%-44%] p = 0.468) revealed no significant difference.

## Discussion

In this retrospective study, we expected HBOT to be a risk factor for VAP. However, this does not seem to be the case, despite the constraints involved (transportation to the hyperbaric chamber, ventilator changes, hyperoxemia). In univariate analysis, immunosuppression, SOFA score, duration of MV, COPD, immunosuppression, neuromuscular blockers, prone position or tracheotomy during hospitalization, reintubation and intra-hospital transport were risk factors for VAP. Only reintubation, intra-hospital transport, use of paralytic agents were independent risk factors in multivariate analysis.

As far as we know, our study is the first to assess the impact of HBOT on the incidence of VAP in mechanically ventilated patients. Our VAP rate is the same as that described in the literature for ICU patients with this degree of severity and these MV durations [22]. Our analysis highlighted a statistical relationship between VAP and several previously described parameters which reinforces its extrinsic validity. The impact on the incidence of VAP in intensive care of neuromuscular-blockers, intra-hospital transport, blood transfusion and reintubations has already been described. Prone position is significantly associated with the occurrence of VAP in their univariate analysis [23]. In a secondary analysis of the PROSEVA study, Ayzac et al. found no significant change in the incidence of VAP in prone patients [24].

Providing oxygen in excess causes tissue damage by producing free radicals responsible for oxidative stress (reactive oxygen species). Hyperoxic Acute Lung Injury is secondary to several mechanisms: pulmonary oedema, hyaline membrane formation, pulmonary arteriole thickening, and deterioration of the ventilation / perfusion fraction by atelectasis. Hyperoxemia also leads to a decrease in muco-ciliary clearance explaining the sensitivity of the lung to bacterial attack during MV [25]. In 2016, Nseir et al. demonstrated that hyperoxemia is an independent risk factor for VAP [8], but the dose effect and the time effect of oxygen toxicity remain unresolved issues.

Hyperbaric hyperoxemia has different physiological consequences from normobaric hyperoxemia. In fact, hyperbaric hyperoxemia, although causing very high arterial and tissue pressures, reduces oxidative stress in many pathologies. Experimental work and clinical studies have shown that HBOT at 2.8 and 3 absolute atmosphere (ATA) effectively opposes the deleterious processes observed during reperfusion, mainly by inhibiting leukocyte adhesion, thus limiting the formation of peroxynitrites [26, 27]. This is because HBOT inhibits the action of beta-2 integrins of polynuclear neutrophils, which blocks leukocyte adhesion and activation leading to the production of superoxide anions [28]. HBOT also prevents the conversion of xanthine dehydrogenase to xanthine oxidase, thereby directly limiting the formation of superoxide anions [29, 30]. Apart from the action on leukocyte adhesion, the anti-inflammatory effect of HBOT has been demonstrated in various animal models, mainly in preconditioning. Generally, HBOT decreases the intensity of the inflammatory response by limiting the production of pro-inflammatory cytokines. These effects are at least partly due to the activation of HIF-1alpha which inhibits the pro-inflammatory activity of the nuclear factor kappa B and also the synthesis of prostaglandins involved in the cyclooxygenase 2 pathway [12, 16]. In addition, the activation of HIF-1alpha by HBOT also increases the expression of heme oxygenase-1 which plays an important role in cellular protection mechanisms to counter the deleterious effects of oxidative stress on DNA. By measuring the plasma concentrations of isoflurane and F2-isoprostanes, Corcoran et al. recently demonstrated that HBOT does not increase oxidative stress [31].

HBOT has effects on aerobic bacteria when the O_2_ pressure exceeds a certain threshold value. Thus partial O_2_ pressures above 1.5 ATA are bacteriostatic in vitro for several aerobic germs such as for *S*. *aureus* or *P*. *aeruginosa*. This effect is variable depending on the germ, the partial pressure of O_2_ and the duration of administration [12]. Patel et al. have demonstrated in animal studies the role of hyperoxemia in the pathophysiology of VAP with *P*. *aeruginosa* [32]. These results were not found for short repeated exposures to HBO [33]. Bacteria generally have a biphasic response to the rise in oxygen pressures with an initial growth stimulation then inhibition for high pressures above 1.5 ATA. This has been demonstrated in particular for *S*. *aureus* [34]. In 1993, Allen and Watt found an alteration in the endothelial function of lung cells subjected to high oxygen concentrations for 12 hours which he did not find under hyperbaric conditions based on the clearance of 5-hydroxytryptamine [35].

Regarding pulmonary functions, repeated hyperbaric oxygen exposure based on the currently used HBOT protocol is safe. In a prospective cohort study published in 2019, Hadanny et al., like Thorsen et al in a previous study, showed that repeated exposures of 60 daily sessions in 2 ATA 100% oxygen had no significant effect on forced vital capacity or pulmonary functions [33, 36]. All this reinforces the hypothesis that, as it is used in hyperbaria, hyperoxemia has no adverse effects on dynamic lung volumes.

Our study suggests that apart from the exposure to the risk factors described, there are no pathophysiological arguments to suspect the accountability of HBOT in the occurrence of VAP.

The incidence of VAP was 23%. The existence of an underlying respiratory history was an independent protective factor. Patients not requiring HBOT were more severe upon admission and older. Initial severity is a known risk for VAP due to longer MV duration [37], however older patients seem less at risk of developing VAP [38].

There a number of limitations to our study. No formal sample size calculation was made and we therefore could not exclude a lack of adequate statistical power to detect an association between HBOT and the occurrence of VAP. In a posteriori power calculation, our study sample size (42 patients with VAP and 140 patients without) enabled us to detect an odds ratio of VAP associated with HBOT of 3.89 (or 0.37 for protective effect), with an 80% power, a two-sided test at 0.05 significance level and by assuming an exposure prevalence (HBOT in patients without VAP) of 70% (as observed in our study). Ours was a retrospective study performed in a single center. We studied the use of HBOT as a whole without differentiating and individualizing the different risk factors linked to its implementation. A prospective study on a wider range of patients is necessary in order to be able to match patients at least on age and severity with an exhaustive collection so confounding factors can be limited.

## Conclusion

The hyperoxemia induced during HBOT does not lead to an increase in the risk of VAP despite the necessary manipulations, transportation and the induced transient hyperoxemia. These results are an encouragement to further our studies on the impact and toxicity of oxygen on our patients. HBOT in the ICU is a safe method as long as it does not lead to longer periods of sedation and mechanical ventilation. Further experimental and clinical investigations are required.

## Supporting information

S1 Table Patient data with / without VAP upon ICU admission. (DOCX)

S2 Table Microbiological isolated with ventilator-associated pneumoniae. (DOCX)

## List of abbreviations

PaO_2_: Arterial partial pressure of oxygen
ICU: Intensive Care Unit
FiO₂: Fraction of inspired oxygen
VAP: Ventilator-acquired pneumonia
HBOT: Hyperbaric oxygen therapy
MV: Mechanical ventilation
SOFA: Sepsis-related organ failure assessment
SAPS: Simplified acute physiology score
COPD: Chronic obstructive pulmonary disease
IQR: Interquartile
BMI: Body mass index
MDR: Multiple drug resistant
ATA: Absolute atmosphere
CO: Carbon monoxide

## References

[1] Panwar R, Hardie M, Bellomo R, et al (2016) Conservative versus Liberal Oxygenation Targets for Mechanically Ventilated Patients. A Pilot Multicenter Randomized Controlled Trial. Am J Respir Crit Care Med 193:43–51. doi: 10.1164/rccm.201505-1019OC 26334785

[2] Kilgannon JH, Jones AE, Shapiro NI, et al (2010) Association between arterial hyperoxia following resuscitation from cardiac arrest and in-hospital mortality. JAMA 303:2165–2171. doi: 10.1001/jama.2010.707 20516417

[3] Yokoyama S, Hifumi T, Kawakita K, et al (2019) Early Hyperoxia in The Intensive Care Unit is Significantly Associated With Unfavorable Neurological Outcomes in Patients With Mild-to-Moderate Aneurysmal Subarachnoid Hemorrhage. Shock Augusta Ga 51:593–598. doi: 10.1097/SHK.0000000000001221 30067563

[4] Palmer E, Post B, Klapaukh R, et al (2019) The Association between Supraphysiologic Arterial Oxygen Levels and Mortality in Critically Ill Patients. A Multicenter Observational Cohort Study. Am J Respir Crit Care Med 200:1373–1380. doi: 10.1164/rccm.201904-0849OC 31513754 PMC6884048

[5] Chu DK, Kim LH-Y, Young PJ, et al (2018) Mortality and morbidity in acutely ill adults treated with liberal versus conservative oxygen therapy (IOTA): a systematic review and meta-analysis. Lancet Lond Engl 391:1693–1705. doi: 10.1016/S0140-6736(18)30479-3 29726345

[6] Dantzker DR, Wagner PD, West JB (1974) Proceedings: Instability of poorly ventilated lung units during oxygen breathing. J Physiol 242:72P 4455844

[7] Hafner S, Beloncle F, Koch A, et al (2015) Hyperoxia in intensive care, emergency, and peri-operative medicine: Dr. Jekyll or Mr. Hyde? A 2015 update. Ann Intensive Care 5:42. doi: 10.1186/s13613-015-0084-6 26585328 PMC4653126

[8] Six S, Jaffal K, Ledoux G, et al (2016) Hyperoxemia as a risk factor for ventilator-associated pneumonia. Crit Care Lond Engl 20:195. doi: 10.1186/s13054-016-1368-4 27334713 PMC4917974

[9] Barrot L, Asfar P, Mauny F, et al (2020) Liberal or Conservative Oxygen Therapy for Acute Respiratory Distress Syndrome. N Engl J Med. doi: 10.1056/NEJMoa1916431 32160661

[10] Helmerhorst HJF, Roos-Blom M-J, van Westerloo DJ, de Jonge E (2015) Association Between Arterial Hyperoxia and Outcome in Subsets of Critical Illness: A Systematic Review, Meta-Analysis, and Meta-Regression of Cohort Studies. Crit Care Med 43:1508–1519. doi: 10.1097/CCM.0000000000000998 25855899

[11] Bessereau J, Aboab J, Hullin T, et al (2017) Safety of hyperbaric oxygen therapy in mechanically ventilated patients. Int Marit Health 68:46–51. doi: 10.5603/IMH.2017.0008 28357836

[12] Jain KK (2017) Textbook of Hyperbaric Medicine, 6th ed. Springer International Publishing

[13] Miller JD, Ledingham IM, Jennett WB (1970) Effects of hyperbaric oxygen on intracranial pressure and cerebral blood flow in experimental cerebral oedema. J Neurol Neurosurg Psychiatry 33:745–755. doi: 10.1136/jnnp.33.6.745 5497875 PMC493587

[14] Fosen KM, Thom SR (2014) Hyperbaric oxygen, vasculogenic stem cells, and wound healing. Antioxid Redox Signal 21:1634–1647. doi: 10.1089/ars.2014.5940 24730726 PMC4175035

[15] Weaver LK (2015) Hyperbaric oxygen treatment for the critically ill patient. Diving Hyperb Med 45:1 25964030

[16] Mathieu D (2006) Handbook on Hyperbaric Medicine. Springer Science & Business Media

[17] Singer M, Deutschman CS, Seymour CW, et al (2016) The Third International Consensus Definitions for Sepsis and Septic Shock (Sepsis-3). JAMA 315:801–810. doi: 10.1001/jama.2016.0287 26903338 PMC4968574

[18] Mathieu D, Marroni A, Kot J (2017) Tenth European Consensus Conference on Hyperbaric Medicine: recommendations for accepted and non-accepted clinical indications and practice of hyperbaric oxygen treatment. Diving Hyperb Med 47:24–32. doi: 10.28920/dhm47.1.24-32 28357821 PMC6147240

[19] Hiner C, Kasuya T, Cottingham C, Whitney J (2010) Clinicians’ Perception of Head-of-Bed Elevation. Am J Crit Care 19:164–167. doi: 10.4037/ajcc2010917 20194613

[20] Infectiologie. In: SRLF. https://www.srlf.org/referentiels/infectiologie/. Accessed 9 Dec 2020

[21] Organisation-Sécurité-Ethique. In: SRLF. https://www.srlf.org/referentiels/organisation-securite-ethique/. Accessed 9 Dec 2020

[22] Koulenti D, Tsigou E, Rello J (2017) Nosocomial pneumonia in 27 ICUs in Europe: perspectives from the EU-VAP/CAP study. Eur J Clin Microbiol Infect Dis Off Publ Eur Soc Clin Microbiol 36:1999–2006. doi: 10.1007/s10096-016-2703-z 27287765

[23] Chastre J, Fagon J-Y (2002) Ventilator-associated pneumonia. Am J Respir Crit Care Med 165:867–903. doi: 10.1164/ajrccm.165.7.2105078 11934711

[24] Ayzac L, Girard R, Baboi L, et al (2016) Ventilator-associated pneumonia in ARDS patients: the impact of prone positioning. A secondary analysis of the PROSEVA trial. Intensive Care Med 42:871–878. doi: 10.1007/s00134-015-4167-5 26699917

[25] Sackner MA, Hirsch JA, Epstein S, Rywlin AM (1976) Effect of oxygen in graded concentrations upon tracheal mucous velocity. A study in anesthetized dogs. Chest 69:164–167. doi: 10.1378/chest.69.2.164 1248269

[26] Kalns J, Lane J, Delgado A, et al (2002) Hyperbaric oxygen exposure temporarily reduces Mac-1 mediated functions of human neutrophils. Immunol Lett 83:125–131. doi: 10.1016/s0165-2478(02)00068-8 12067761

[27] Thom SR (1993) Functional inhibition of leukocyte B2 integrins by hyperbaric oxygen in carbon monoxide-mediated brain injury in rats. Toxicol Appl Pharmacol 123:248–256. doi: 10.1006/taap.1993.1243 8248932

[28] Thom SR (1990) Antagonism of carbon monoxide-mediated brain lipid peroxidation by hyperbaric oxygen. Toxicol Appl Pharmacol 105:340–344. doi: 10.1016/0041-008x(90)90195-z 2219124

[29] Thom SR (1992) Dehydrogenase conversion to oxidase and lipid peroxidation in brain after carbon monoxide poisoning. J Appl Physiol Bethesda Md 1985 73:1584–1589. doi: 10.1152/jappl.1992.73.4.1584 1447108

[30] Thom SR, Bhopale VM, Velazquez OC, et al (2006) Stem cell mobilization by hyperbaric oxygen. Am J Physiol Heart Circ Physiol 290:H1378–1386. doi: 10.1152/ajpheart.00888.2005 16299259

[31] Corcoran T, Ting S, Mas E, et al (2017) Hyperbaric oxygen therapy is not associated with oxidative stress assessed using plasma F2-isoprostanes and isofurans. Prostaglandins Leukot Essent Fatty Acids 127:16–19. doi: 10.1016/j.plefa.2017.10.003 29156153

[32] Patel VS, Sitapara RA, Gore A, et al (2013) High Mobility Group Box-1 mediates hyperoxia-induced impairment of Pseudomonas aeruginosa clearance and inflammatory lung injury in mice. Am J Respir Cell Mol Biol 48:280–287. doi: 10.1165/rcmb.2012-0279OC 23087050 PMC3604087

[33] Thorsen E, Aanderud L, Aasen TB (1998) Effects of a standard hyperbaric oxygen treatment protocol on pulmonary function. Eur Respir J 12:1442–1445. doi: 10.1183/09031936.98.12061442 9877506

[34] Tsuneyoshi I, Boyle WA, Kanmura Y, Fujimoto T (2001) Hyperbaric hyperoxia suppresses growth of Staphylococcus aureus, including methicillin-resistant strains. J Anesth 15:29–32. doi: 10.1007/s005400170048 14566544

[35] Allen MC, Watt SJ (1993) Effect of hyperbaric and normobaric oxygen on pulmonary endothelial cell function. Undersea Hyperb Med J Undersea Hyperb Med Soc Inc 20:39–48 8471959

[36] Hadanny A, Zubari T, Tamir-Adler L, et al (2019) Hyperbaric oxygen therapy effects on pulmonary functions: a prospective cohort study. BMC Pulm Med 19:148. doi: 10.1186/s12890-019-0893-8 31409407 PMC6693142

[37] Martin-Loeches I (2020) Current Concepts in Community and Ventilator Associated Lower Respiratory Tract Infections in ICU Patients. Antibiotics 9:. doi: 10.3390/antibiotics9070380 32635601 PMC7399936

[38] Dananché C, Vanhems P, Machut A, et al (2018) Trends of Incidence and Risk Factors of Ventilator-Associated Pneumonia in Elderly Patients Admitted to French ICUs Between 2007 and 2014. Crit Care Med 46:869–877. doi: 10.1097/CCM.0000000000003019 29432348

