# Supplementary material for: Hyperbaric hyperoxemia as a risk factor for ventilator-acquired pneumonia?
Source: PLoS One. 2021 Jun 23;16(6):e0253198. doi: 10.1371/journal.pone.0253198 (PMC8221473; doi:10.1371/journal.pone.0253198)
Supplement: S1 Table — (DOCX) [file pone.0253198.s001.docx]

**S1 Table. Patient data with / without VAP upon ICU admission**

|  | **No VAP (n = 140)** | **VAP (n = 42)** | ***p*** |
| --- | --- | --- | --- |
| Male gender | 88 (63%) | 32 (76%) | 0.11 |
| Age (*years*) | 59,5 [46-69] | 60 [49-67] | 0.94 |
| **Comorbidities (*%*)** |  |  |  |
| Diabetes | 47 (34%) | 14 (33%) | 0.98 |
| COPD | 29 (21%) | 3 (7%) | **0.043** |
| Heart failure | 20 (14%) | 6 (14%) | 0.96 |
| Kidney failure | 13 (9%) | 4 (9%) | 1 |
| Cirrhosis | 5 (4%) | 5 (12%) | 0.052 |
| Immunosuppression | 9 (6%) | 8 (19%) | **0.029** |
| Neoplasia | 17 (12%) | 6 (14%) | 0.71 |
| Weight (*kg)* | 80 [69–92] | 80 [70–94] | 0.84 |
| BMI (*kg.m^-2^*) | 29 [25–36] | 27 [23–32] | 0.15 |
| **Reason for admission (*%*)** |  |  |  |
| Medical (vs surgical) | 85 (61%) | 32 (76%) | **0.066** |
| Shock | 68 (49%) | 18 (43%) | 0.52 |
| Sepsis | 58 (41%) | 15 (36%) | 0.51 |
| Respiratory failure | 19 (14%) | 9 (21%) | 0.22 |
| Drug intoxication | 3 (2%) | 2 (5%) | 0.33 |
| CO poisoning | 6 (4%) | 1 (2%) | 1 |
| Self-attempted hanging | 28 (20%) | 7 (17%) | 0.63 |
| Gas embolism | 5 (4%) | 5 (12%) | 0.052 |
| Neurological failure | 28 (20%) | 8 (19%) | 0.89 |
| Cardiac arrest | 31 (22%) | 7 (17%) | 0.44 |
| Acute cardiac failure | 4 (3%) | 1 (2%) | 1 |
| Cellulitis | 54 (39%) | 9 (21%) | **0.041** |
| **Severity scores** |  |  |  |
| SOFA | 7 [5-9] | 8 [5-11] | 0.07 |
| SAPS II | 59 [45-70] | 61 [44-75] | 0.42 |
| Prior antibiotic therapy | 26 (19%) | 7 (16%) | 0.73 |

Results are expressed in numbers (%) for categorical variables and in median (IQR) or mean ± standard deviation for quantitative variables. Exposure to risk factors was collected until the onset of VAP or until extubation. VAP: Ventilator acquired pneumonia, ICU: Intensive care unit, COPD: chronic obstructive pulmonary disease, BMI: body mass index, CO: Carbon monoxide, SOFA: sequential organ failure assessment, SAPS II: simplified acute physiological score.
