# Supplementary material for: Hyperbaric hyperoxemia as a risk factor for ventilator-acquired pneumonia?
Source: PLoS One. 2021 Jun 23;16(6):e0253198. doi: 10.1371/journal.pone.0253198 (PMC8221473; doi:10.1371/journal.pone.0253198)
Supplement: S2 Table — (DOCX) [file pone.0253198.s002.docx]

**S2 Table. Microbiological isolated with ventilator-associated pneumoniae**

| **Pathogens** | **Number (%)** |
| --- | --- |
| **Gram-negative bacilli** | **36 (77%)** |
| *Pseudomonas aeruginosa* | 6 (13%) |
| *Stenotrophomonas maltophilia* | 1 (2%) |
| *Escherichia coli* | 3 (6%) |
| Klebsiella sp. | 10 (21%) |
| Proteus sp. | 4 (8%) |
| Enterobacter sp. | 5 (11%) |
| Others | 7 (15%) |
| **Gram-positive cocci** | **11 (23%)** |
| Methicillin-responsive *Staphylococcus aureus* | 7 (15%) |
| *Streptococcus pneumoniae* | 3 (6%) |
| **MDR pathogens** | **11 (23%)** |
| **Other** | **1 (2%)** |
| *Enterovirus* | 1 (2%) |
